# Dynamic water patterns change the stability of the collapsed filter conformation of the KcsA K^+^ channel

**DOI:** 10.1371/journal.pone.0186789

**Published:** 2017-10-19

**Authors:** Di Wu

**Affiliations:** Department of Physiology and Biophysics, School of Life Sciences, Fudan University, Shanghai, People’s Republic of China; Zhejiang University Life Science Institute, CHINA

## Abstract

The selectivity filter of the KcsA K^+^ channel has two typical conformations—the conductive and the collapsed conformations, respectively. The transition from the conductive to the collapsed filter conformation can represent the process of inactivation that depends on many environmental factors. Water molecules permeating behind the filter can influence the collapsed filter stability. Here we perform the molecular dynamics simulations to study the stability of the collapsed filter of the KcsA K^+^ channel under the different water patterns. We find that the water patterns are dynamic behind the collapsed filter and the filter stability increases with the increasing number of water molecules. In addition, the stability increases significantly when water molecules distribute uniformly behind the four monomeric filter chains, and the stability is compromised if water molecules only cluster behind one or two adjacent filter chains. The altered filter stabilities thus suggest that the collapsed filter can inactivate gradually under the dynamic water patterns. We also demonstrate how the different water patterns affect the filter recovery from the collapsed conformation.

## Introduction

It is well known that buried waters can fill the vacancies in protein structures and affect their stabilities [1–4]. Due to hydrogen bonding to protein atoms, these water molecules function like protein’s intrinsic structural elements, stabilizing protein’s local conformation that may otherwise deform. Recently, this role of water has been found in the KcsA K^+^ channel [5, 6].

**Fig 1:**
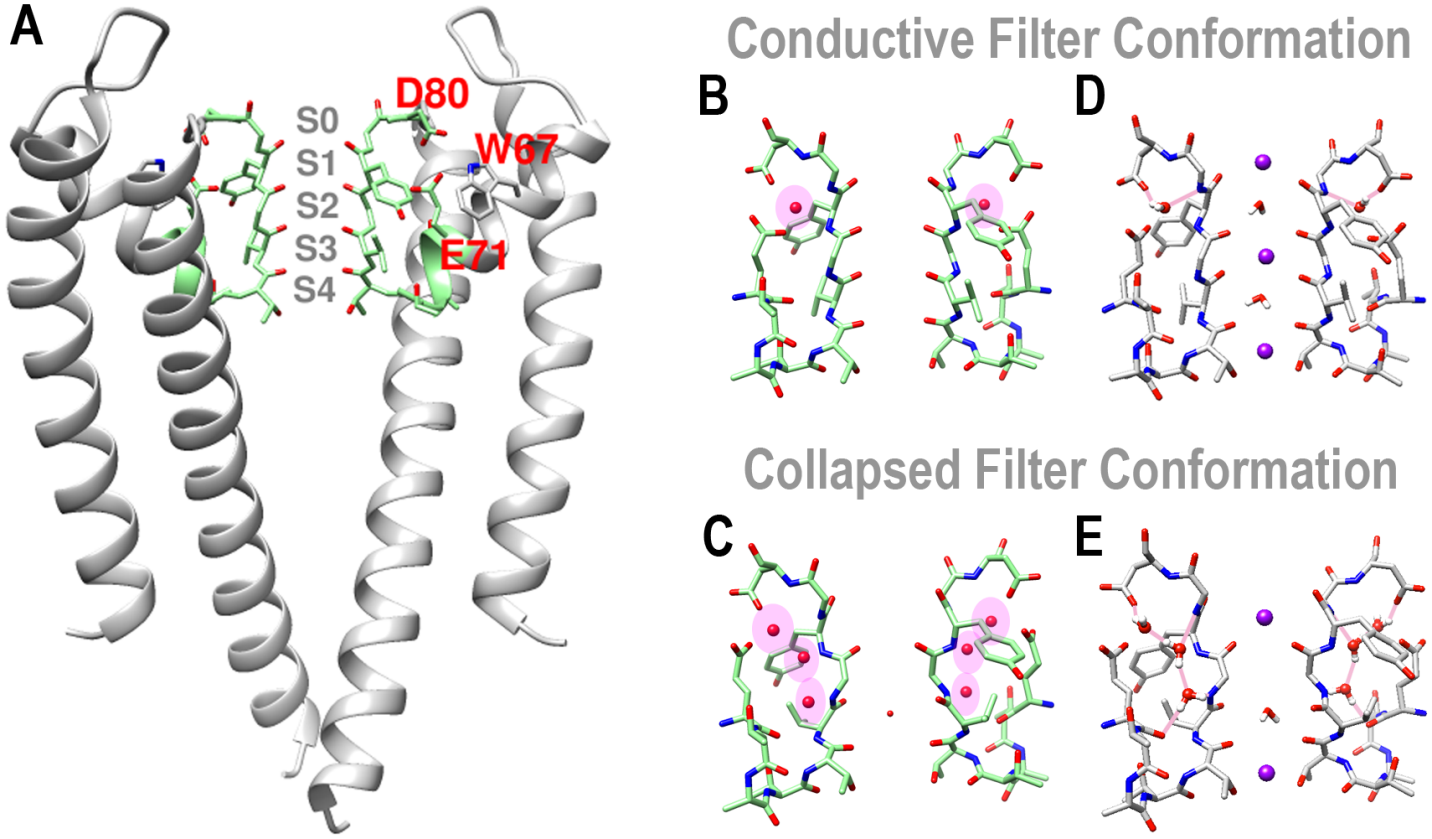
The KcsA K^+^ channel structure. (A) Crystal structure of the KcsA channel (PDB ID: 1K4C). (B) The conductive filter conformation (PDB ID: 1K4C). (C) The collapsed filter conformation (PDB ID: 1K4D). The water molecules behind the selectivity filters are highlighted in the pink ovals. Equilibrating the structures of (B) and (C), the simulation results are shown in (D) and (E), where the hydrogen bonds connecting the percolated waters and the presented protein atoms are shown. For clarity, only two chains of the channel structures are shown.

The KcsA K^+^ channel is a tetrameric transmembrane protein that regulates ion conduction through membranes using two functional domains—the inner helix bundle gate and the selectivity filter (Fig 1A). Crystal structures show that the selectivity filter has two typical conformations [7] called the conductive and the collapsed conformations, respectively (Fig 1B and 1C). Ions can go through a conductive filter but visually cannot go through a collapsed one because the S2 site is too narrow to accommodate an ion. Thus the collapsed filter is assumed to impede the ion permeation. Reversely, the channel resumes the ability to conduct ions when the collapsed filter switches to the conductive conformation that can represent the channel recovery. The conformational change of the selectivity filter in the KcsA channel is well characterized in several NMR studies [8–10]. And this conformational change depends on many factors. A conductive filter prefers a high K^+^ concentration [7] and a closed inner gate [11, 12], whereas a collapsed one prefers a low K^+^ concentration [7] and an open inner gate [11, 12]. In addition, this conformational change is regulated directly by the filter residues [13], and indirectly by the pore helix residues [14–17].

In the KcsA crystal structures, an interspace behind the filter is observed, which is delineated by E71, D80 and the filter residues. Due to the rearrangements of the local residues, this interspace contracts in the conductive conformation and expands a little in the collapsed conformation, which enables the extracellular water molecules to permeate behind the filter chains. And these water molecules can enhance the filter stability. To distinguish these water molecules from those permeating normally through the filter, we call them the *percolated* waters in this paper. The water percolating sites behind the selectivity filter of the KcsA channel have been found in several experimental studies. In the solid-state NMR studies, the NMR spectra show one buried water behind a conductive filter chain [6, 18] and several buried waters behind a collapsed filter chain [6]. These are consistent with those found in the crystal structures, where a single water site is found behind a conductive filter chain (PDB ID: 1K4C, Fig 1B), and up to three water sites spread vertically behind a collapsed filter chain (PDB ID: 1K4D, Fig 1C). Assigning hydrogen atoms to the crystal structures and equilibrating them in the molecular dynamics simulations, a well-organized hydrogen-bond network is revealed (Fig 1D and 1E). Clearly, in the collapsed filter conformation, the percolated waters are indispensable in this tightly bonding network that can enhance the local structural stability.

Ostmeyer et al. have examined the stability of such a collapsed filter structure (Fig 1C) that the collapsed filter can hardly switch back to the conductive conformation bearing these percolated waters because the free-energy penalty upon the filter recovery from this structure is almost 25 kcal/mol [5]. Their work provides the novel and insightful viewpoint on the percolated waters to the stability of the collapsed filter conformation. If the percolated waters can lock the collapsed filter inactivated, then when and how do these water molecules move in? Should all the water sites be filled (as shown in Fig 1C) to inactivate a filter? If the water patterns become different, can they still affect the filter stability? And how can the different water patterns influence the protein conformational change during the process when the filter recovers from the collapsed conformation?

In this paper, we try to address these questions using the molecular dynamics simulations. We have observed the spontaneous formation of the collapsed filter of the KcsA K^+^ channel in the simulations when the K^+^ ions inside the filter move out, leaving the S2 and S3 sites unoccupied by ions. In the subsequent simulations of equilibrating the collapsed filter structures, we occasionally observe water molecules percolating behind the collapsed filter chains. They scatter behind the four filter chains, forming the irregular patterns. Experiments show that the KcsA channel inactivates and recovers both over seconds [19]. During these processes, water molecules may percolate behind the collapsed filter or move out gradually. To circumvent the limitation of the simulation length, we specifically design the different water patterns behind a collapsed filter and study the filter stability under these water patterns. Many factors can influence the collapsed filter stability, however, the effects of the percolated waters are noticeable. They are sufficient to vary the strength of a collapsed filter, changing it from an easily conductive to a nearly nonconducting filter. Combining the simulation results, we hypothetically draw a picture of the gradually stabilized filter under the different water patterns, which subsequently can affect the filter recovering from the collapsed conformation.

## Methods

The simulations were conducted by the NAMD molecular simulation package [20] and the results were analyzed by VMD [21] and displayed by Chimera [22]. We used PDB files 3F7V and 1K4D for the collapsed filter structures and 1K4C for the conductive filter structure for the KcsA K^+^ channel. The CHARMM force field parameters were used, c22 for proteins [23] with CMAP corrections included [24], and c27 for lipids [25, 26]. Standard parameters for ions were used, and the modified parameters for carbonyl oxygen and cation interactions were included [27–29]. Each channel structure was embedded in the POPC lipid bilayer and then put in a box filled with the TIP3P water molecules. The KCl concentration was set to 0.15 M in each simulation box. In each structure, the residue E71 of each chain was protonated, which was reported as an important factor in the KcsA channel simulations [30]. The all-atom molecular dynamics simulations were conducted under the constant temperature of 310 K and constant pressure of 1 atm. The temperature was controlled by the Langevin thermostat with the damping coefficient of 1 ps^-1^, and the pressure was controlled by the Nose-Hoover Langevin piston with the period of 200 ps and the damping time constant of 50 ps. Particle Mesh Ewald method was used for the electrostatic interactions. The nonbonded interactions were truncated at 12 Å and the switching functions were applied starting at 10 Å. Bonds involving the hydrogen atoms were constrained using the SHAKE algorithm. Simulations were conducted with the time step of 2 fs. The r-RESPA multiple time step scheme was used, where the short-range nonbonded forces were updated every 2 fs and the long-range electrostatic forces were updated every 4 fs.

The filter structure in the 3F7V file has the collapsed conformation. After initial equilibrations, we pulled the K^+^ ion at the S4 site in the outward direction with the constant force of 3.5 nN applied every 10 fs until it moved to the S2 site that switched the collapsed filter to the conductive conformation. This structure was equilibrated for 3 ns by restraining the structure containing the conductive filter conformation (residues T74 to L81) with the harmonic restrains where the force constant was set to 1 kcal·mol^-1^·Å^-2^. Then we placed three K^+^ ions at the S0, S2, and S4 sites and two water molecules at the S1 and S3 sites in this conductive filter. After 2 ns equilibration, we pulled K^+^ upward out of the selectivity filter of the 3F7V channel structure, and observed that in some case the water molecule percolated deeply behind the collapsed filter following the filter conformational switch process. Using these results, we placed water molecules in the different patterns behind the collapsed filter chains. All the patterns were randomly designed by arbitrarily placing one to three water molecules over the three percolating sites behind each filter chain. After equilibrations, we selected eight patterns for the study and denoted them as models M1-M8 in the paper, where K^+^ ions were placed in the S1 and S4 sites in each collapsed filter. Models M9 and M10 directly used the PDB files 1K4D and 1K4C as the initial structures, where the K^+^ ions and the water molecules were placed in the filters as shown in Fig 1E and 1D, respectively. The ten models were equilibrated further for 5–10 ns. And in some cases, we applied the extra force to restrain the K^+^ ions in the S1 and S4 sites inside the filter. From the simulations examined, we observed that the patterns composed of a small number of water molecules changed, typically more water molecules moved behind the collapsed filter bearing few percolated waters. This can happen over several nanoseconds. In addition, the ions and the water occupancies inside the selectivity filter may also change. Thus to compare the model structures as designed, we used only short equilibration runs in the analyses (5–10 ns).

To compare the filter stabilities of the ten models, the K^+^ ions inside the filter were pulled upward through the filter under the constant force (ranged from 2 to 4.5 nN) applied every 10 fs. The force was turned off when the ion moved out of the filter, defined as the location of the ion above the center of Cα of D80. The procedure conducted under each force value was repeated six times for each model system. And the average value of ln(I_app_/I_app,max_) versus the force curves were fitted using the natural logarithm of the equation:

$$\frac{{\text{I}}_{app}}{{\text{I}}_{app,\text{max}}}=\frac{1}{1+e^{-\beta \cdot (F-F_{1/2})\cdot d}}$$ (1)

Where *F*_*1/2*_ describes the force required when the current I reaches one half of the maximum value, and *d* is the apparent distance that the ion moves along under the given force. *β* equals 1/*k*_B_T, where *k*_B_ is Boltzmann’s constant and T is the Kelvin temperature. Note that the curves fitted by Eq (1) cannot be compared to the experimental data due to two reasons. First, the forces used in the simulations are large and are applied to the filter ions every 10 fs (in order to relax the local structure), which is different from the real experiment. Second, we only study the single ion permeation event at the initial stage of the filter recovering from the collapsed conformation whereas in experiment multiple ions can continuously go through the filter after the filter begins to conduct ions. Thus the current I studied here has the different meaning than that measured in the experiment, so we use the symbol I_app_. Therefore, the curves fitted by Eq (1) are used only to compare the stabilities of the different collapsed filter conformations that undergo the procedures in the same condition.

## Results

### Water molecules percolate dynamically behind a collapsed filter, forming different patterns

**Fig 2:**
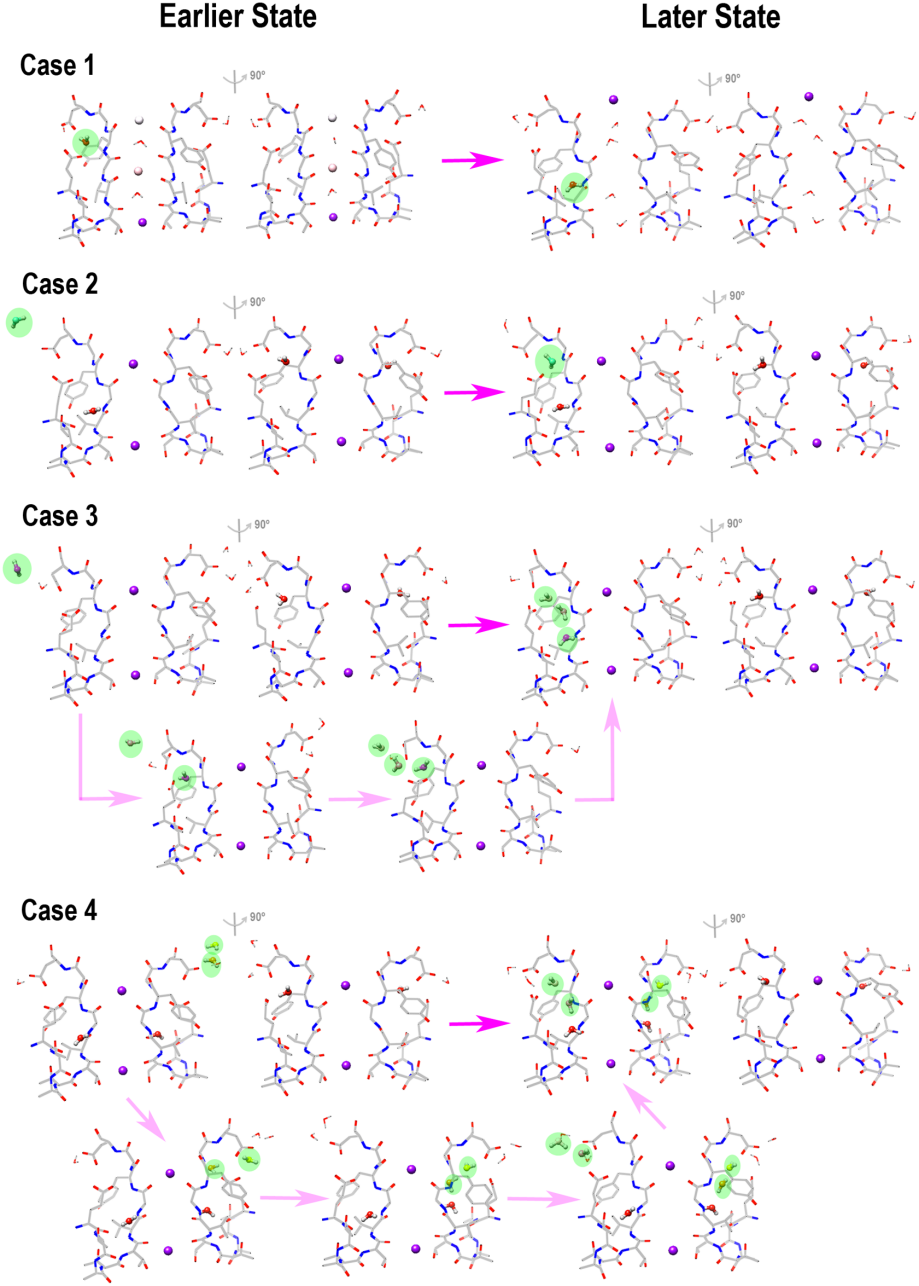
Processes of water molecules percolating behind the selectivity filter of the KcsA channel. In Case 1, one water molecule percolates deeply as the conductive filter switches to the collapsed conformation. Cases 2–4 show the different processes of the water molecules percolating behind the collapsed filter chains. In addition to the earlier state and the later state filter structures, Cases 3 and 4 also show the step-by-step procedures of the percolating events. The percolating waters are highlighted in the green ovals. Here each case starts from a different initial configuration. The two structures of Case 1 are selected at the simulation time of 0 ns and 0.334 ns, respectively. The two structures of Case 2 are selected at the simulation time of 4.040 ns and 4.082 ns, respectively. The four structures of Case 3 are selected at the simulation time of 6.430 ns, 6.756 ns, 6.970 ns, and 7.648 ns, respectively. The five structures of Case 4 are selected at the simulation time of 4.072 ns, 4.260 ns, 4.634 ns, 5.802ns, and 6.486 ns, respectively. The enlarged figures including these structures and several more structures of each case are shown in S1 Fig.

In the KcsA channel, water molecules start to percolate deeply behind the filter after the conductive filter switches to the collapsed conformation, because the rearrangement of the local structures behind the selectivity filter can make extra room for the deeply percolated waters. We observe this in the simulation. In Case 1 (Fig 2), after the short equilibration of 20 ps, the K^+^ ion at the S4 site is pulled upward through the filter under the constant force of 3.5 nN applied every 10 fs. After the conductive filter switches to the collapsed conformation, the percolated water (located in the upper site) moves deeply behind the filter. In other cases, when equilibrating several collapsed filter conformations with the designed water patterns, we observe more water molecules percolating behind the filter but in the different patterns. They enter behind the filter either separately (Case 2 in Fig 2) or in groups (Case 3 in Fig 2), and sometimes chain by chain (Case 4 in Fig 2). These results demonstrate that water molecules percolate dynamically behind the collapsed filter, forming the different patterns. The crystal structure shows the fully occupied water pattern (Fig 1C), which is possibly formed through a step-by-step procedure. Therefore, the filter inactivation process is associated with the dynamic change of the water patterns behind the filter. The durations of these water patterns are short as seen in S1 Fig that the initial water patterns are replaced by the new patterns over several nanoseconds, so the patterns with few percolated waters may appear only at the initial stage of the filter inactivation process. Because these water patterns can change from time to time and from case to case, in order to know their influences to the collapsed filter stability, we have designed and selected several water patterns for the study.

### Some percolated waters seem stationary whereas others are fluid

**Fig 3:**
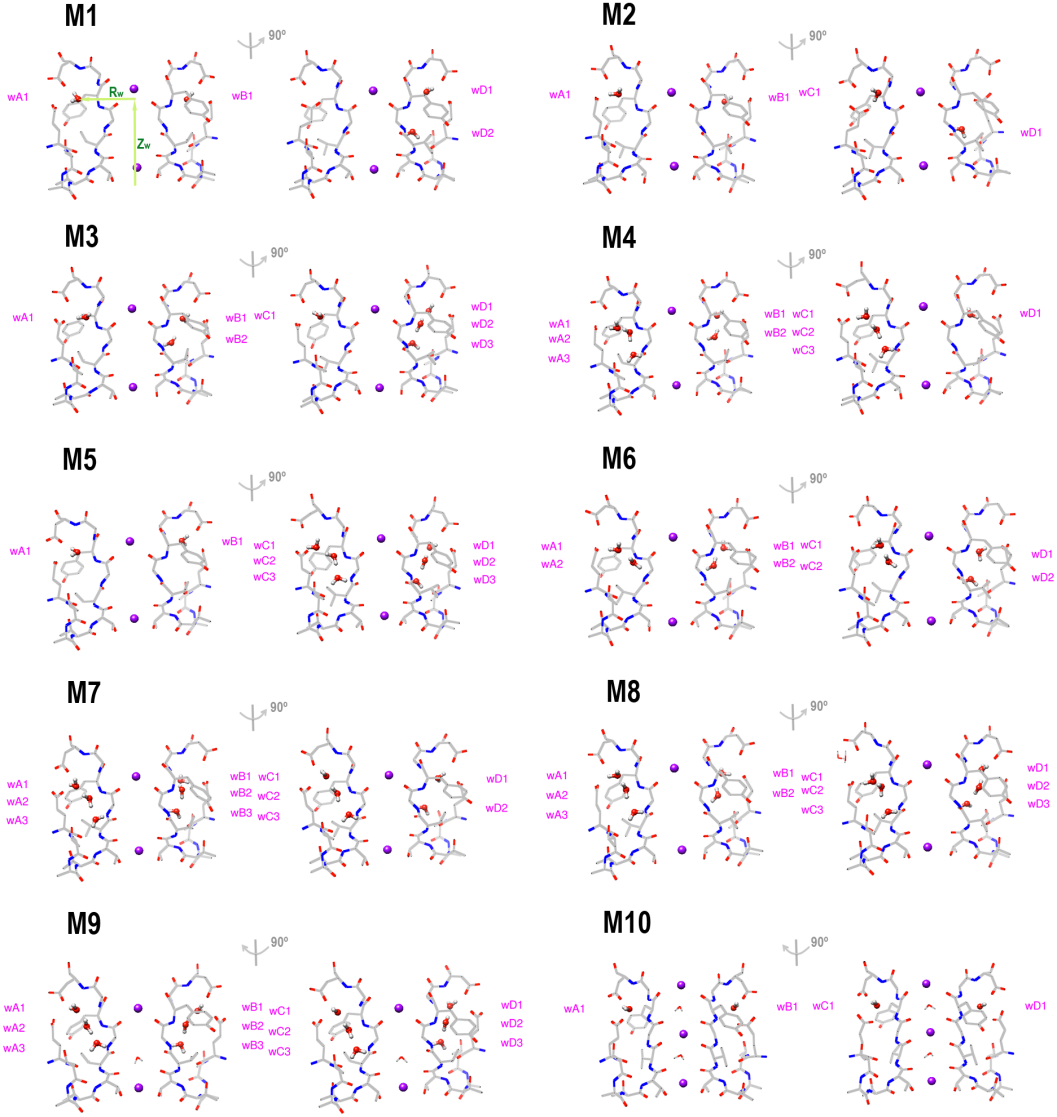
Ten models having the different water patterns behind the KcsA filter chains. The percolated waters are labeled in each model. In the collapsed filter conformations, models M1-M8 are built based on the PDB file 3F7V and M9 is based on the PDB file 1K4D. The conductive filter conformation, model M10, is based on the PDB file 1K4C. In model M8, the bond of D80 and E71 behind chain C is broken and filled with water molecules. R_w_ and Z_w_ are drawn for the water wA1 in model M1, where R_w_ is the horizontal distance of the water oxygen to the central axis of the filter and Z_w_ is the vertical distance of the water oxygen to the center of Cα of residue T74 of the four chains. The enlarged figures are shown in S2 Fig.

We study ten models (Fig 3, enlarged figures shown in S2 Fig), eight collapsed filters bearing the variously designed water patterns (models M1-M8 based on PDB file 3F7V), one collapsed filter having the percolated waters occupying all sites (model M9 based on PDB file 1K4D), and one conductive filter having four percolated waters (model M10 based on PDB file 1K4C). Here we use both PDB structures 3F7V (having an open inner helix bundle gate) and 1K4D (having a closed inner helix bundle gate) in the study. Experiments show that an open inner gate and a collapsed filter conformation are correlated [11, 12]. Because the 3F7V structure has an open inner gate, we use it to study the properties of the collapsed filter conformation. For comparison, we also study the collapsed filter in the 1K4D structure that has a closed inner gate.

**Fig 4:**
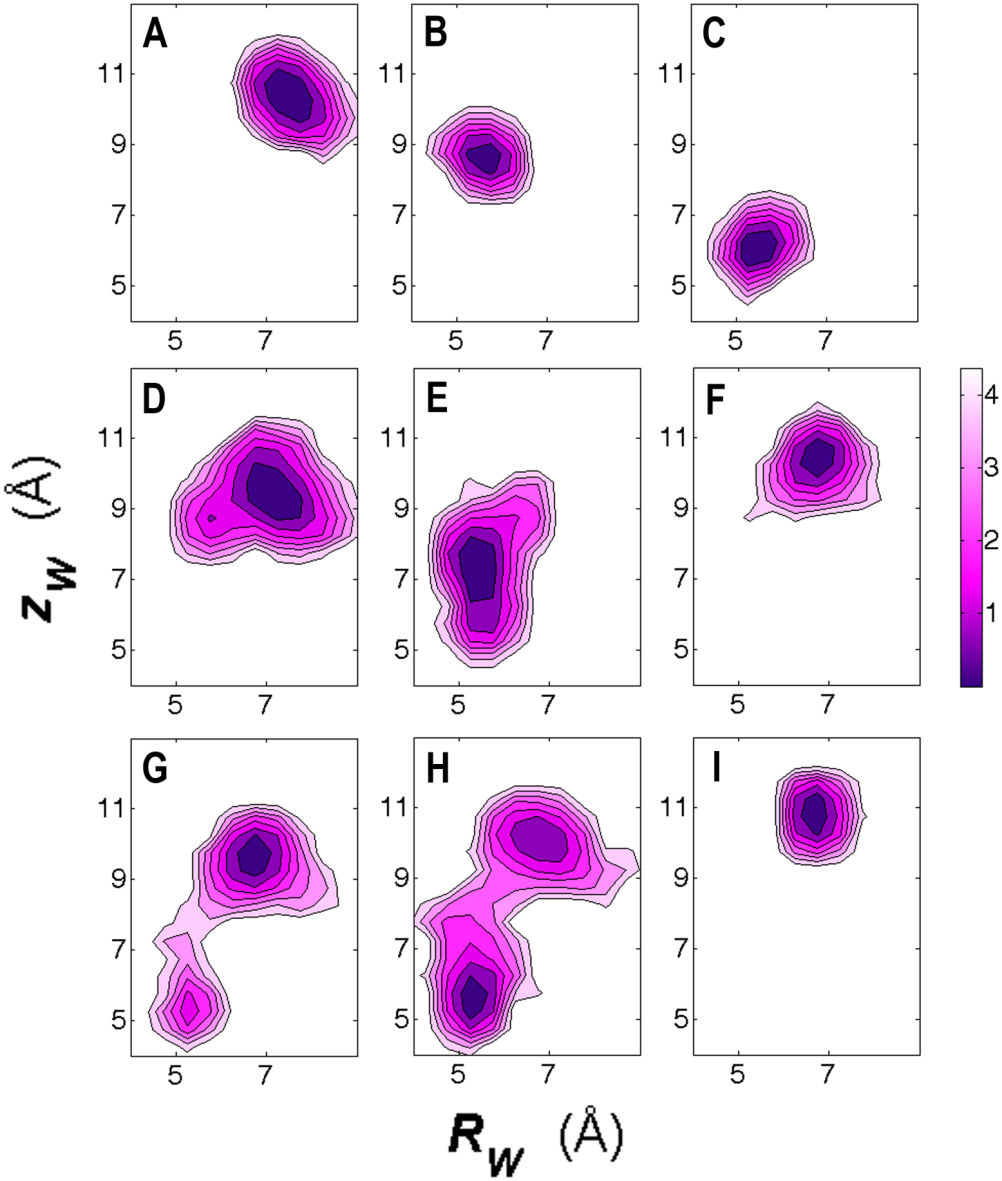
Free-energy maps of the selected water locations in the ten models. (A)-(C) show the locations of the waters wC1, wC2, and wC3 of model M5, respectively. (D) and (E) show the locations of the waters wB1 and wB2 of model M6, respectively. (F) and (G) show the locations of the waters wA1 and wB1 of model M5, respectively. (H) shows the location of the water wA1 of model M2. Thus, (A)-(C) show the free-energy maps when three water molecules percolate behind one filter chain; (D) and (E) show the maps of two water molecules behind one chain; (F)-(H) show three maps when one water molecule is there. (F)-(H) show the versatile locations of the water molecule, which may depend on the water patterns of the adjacent chains. (I) shows the location of the water wC1 of model M10 that has a conductive filter. The color bar has the unit of kcal/mol. Z_w_ and R_w_ are defined in Fig 3.

Equilibrating these structures and plotting the free-energy map for each water molecule, we easily notice their similarities and differences. For the short equilibration time examined (5–10 ns), water motions behind the collapsed filters can be categorized into three general groups based on the number of water molecules behind one filter chain (Fig 4). The water molecules seem stationary when they fill all three sites behind one filter chain. The well-organized hydrogen bond network greatly restricts their movements (Fig 4A–4C). When two water molecules percolate behind one filter chain, the top one moves between the top two sites and prefers the upper site (Fig 4D), whereas the bottom one moves freely between the lower two sites (Fig 4E). When only one water molecule percolates behind one chain, it can remain in the upper site (Fig 4F) or gradually change its preference and occupy all three sites (Fig 4G and 4H). This may depend on the water patterns of the adjacent chains. The water molecules behind a conductive filter move less (Fig 4I). The upper site seems to be the only location for the percolated water, and the free-energy map shows the restricted region for the movement.

### Water patterns significantly change the collapsed filter stability

**Fig 5:**
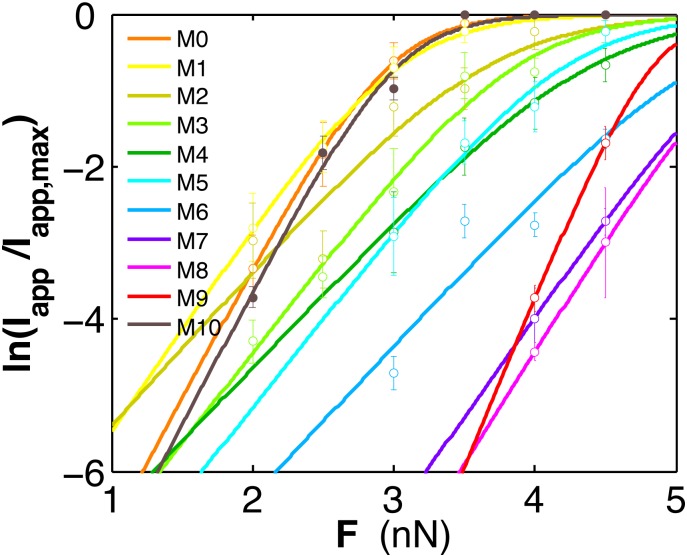
Filter stability under the different water patterns. Logarithm of the normalized current is plotted as a function of the force applied to the filter ions of the ten models. The extra model M0 (having a collapsed filter conformation) contains no percolating water.

The filter stabilities of the ten models are compared in Fig 5. For comparison, we also include an extra model M0 that has no water occupying any of the percolating sites. Note these curves can be used to compare the filter stabilities but cannot be used to compare with the experimental data (explained in Methods section). The strengths of the collapsed filters of models M0 and M1 are comparatively small as that of the conductive filter (model M10), whereas models M2 to M8 show the increased filter stabilities. These results correlate well with the common view that the collapsed filter can be stabilized with the increasing number of the percolating waters. In addition, we also recognize several special features of the water patterns. In models M1-M9, filters having the asymmetrically distributed water molecules (M3, M4) are less stable than those having the uniformly distributed water molecules (M6). And the filter stability is relatively high if a large number of water molecules distribute diagonally (M5). The filter stability is greatly enhanced when all the water sites are fully (M9) or nearly fully occupied (M7, M8). Note that one E71-D80 bond is missing in model M8, but this does not reduce its high filter stability. The relatively high stabilities of the filters (M6-M9) imply that water molecules need not fill all the vacancies to inactivate a collapsed filter.

Although models M1-M8 show varied filter stabilities, they may only represent the intermediate-stage structures that occur at the initial stage of the long inactivation process (lasting over seconds) but not the inactivated-state structures, because all of them have been equilibrated only for a short time (5–10 ns). In fact, over the long inactivation period, water molecules can continuously percolate behind the collapsed filter and occupy all the vacancies, forming a pattern like that shown in model M9. Ostmeyer et al. have shown that the percolated waters can occupy all sites behind the filter in the microsecond time scale simulations (Supporting information in Ref. [5]). Here, only model M9 may resemble the inactivated state structure because the initial channel structure is directly taken from the PDB file with the water pattern as observed in the crystal structure (note that model M9 has a closed inner gate). Although not representing the inactivated state structures, models M7 and M8 show the high filter stabilities resembling that of model M9. This means that the collapsed filter can be stabilized quickly when a lot of water molecules percolate behind the filter. And this stabilization process can be completed in a short time because the time required for water molecules percolating all the sites is typically within microsecond according to the results of Ostmeyer et al. [5]. Therefore, the results presented in Fig 5 imply that the initially formed collapsed filter (with few percolating waters) may not be stable, but it can be stabilized quickly (with a lot of percolating waters).

### Water patterns influence the filter recovery from the collapsed conformation

**Fig 6:**
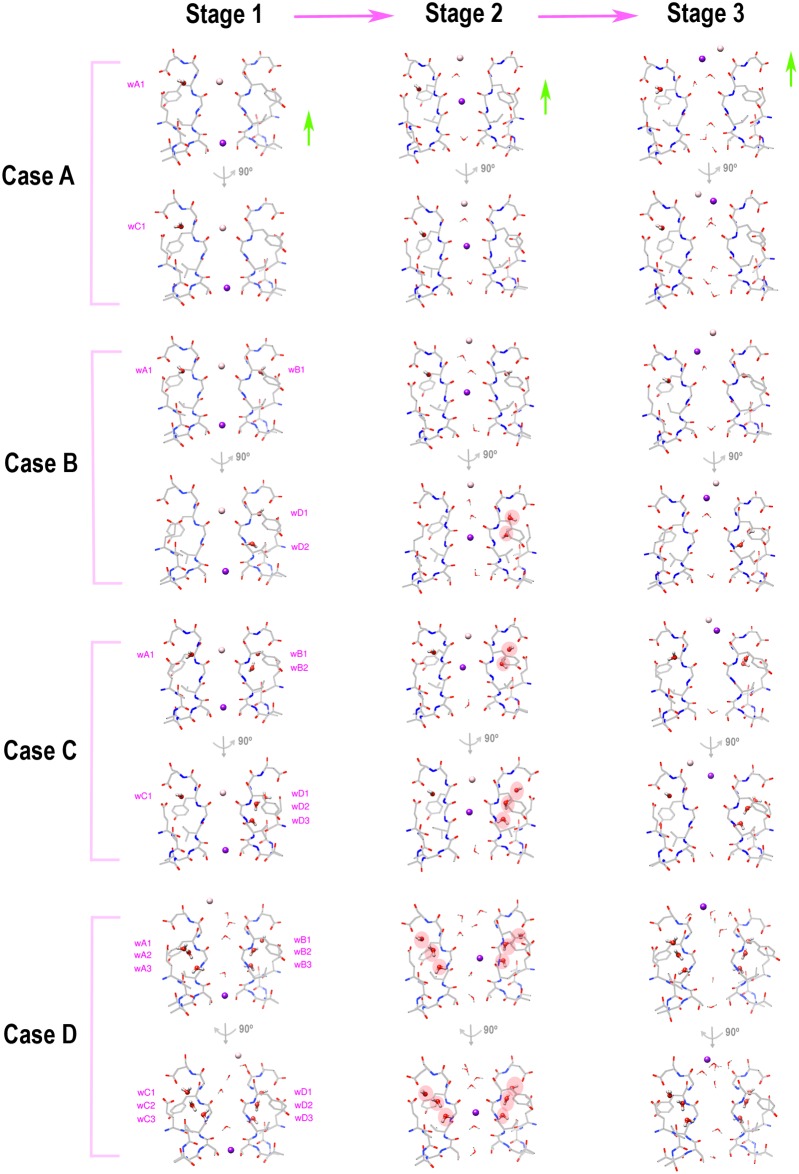
Initial step of the filter recovery affected by the different water patterns. Four cases are selected to illustrate the conformational change of the filter as the K_4_^+^ ion moves through the collapsed filter. In Case A, two percolating waters locate in the upper sites behind chains A and C, respectively. Cases B, C, and D represent models M1, M3, and M9, respectively. Initially (Stage 1), all filters are in the collapsed conformations. As the ion moves to the S2 site (Stage 2), the collapsed filters switch to the conductive conformation (Case A), the nearly conductive conformation (Case B), the partially conductive conformation (Case C), and the nearly collapsed conformation (Case D), respectively. When the ion moves out of the filter (Stage 3), all the filters switch back to the collapsed conformations again. The percolated waters that prevent (or tend to prevent) the filter chains from switching back to the conductive conformations are highlighted in the red ovals. The three structures of Case A are selected at simulation time of 0 ps, 35.24 ps, and 67.08 ps, respectively. Structures of Case B are selected at 0 ps, 15.04 ps, and 21.16 ps, respectively. Structures of Case C are selected at 0 ps, 6.56 ps, and 10 ps, respectively. Structures of Case D are selected at 2 ps, 113.04 ps, and 117 ps, respectively. The enlarged figures are shown in S3 Fig. The initial configuration of Case D is obtained from a separate simulation shown in S3 Fig Part D.

We simulate the initial step of the filter recovery, and select four typical cases to illustrate this process (Fig 6, enlarged figures shown in S3 Fig). Here, recovery simply means the filter recovering from the collapsed conformation to conduct ions, not recovering from the inactivated state because these are not the inactivated state structures. The four cases in Fig 6 demonstrate the concerted movements of the percolated waters and the spontaneous response of the filter chains upon the K_4_^+^ ion (the K^+^ ion initially at the S4 site inside the filter) crossing a collapsed filter. Here Case A is included to show the effect of a special water pattern (two percolating waters, each behind one chain). This structure is selected directly from one of the initial designs of the water patterns, which is not equilibrated further because more water molecules can percolate behind the filter if equilibrating for a long time. Therefore, this temporary structure is not included in the ten models studied in Fig 3, but it is included here simply for comparison. Cases B-D represent models M1, M3, and M9 respectively. To show the filter conformational change process, the forces (3 nN in Case A, 3.5 nN in Cases B and C, and 4.5 nN in Case D) are applied to the filter ions in the upward direction every 10 fs. The initial configuration of Case D uses the result from a separate simulation (lasting for 94 ps, structures shown in S3 Fig Part D) where the filter K^+^ ions are pulled upward under the force of 4.5 nN applied every 10 fs. In that simulation, only K_1_^+^ (K^+^ ion initially at the S1 site) moves out of the filter. In Case D, after a short equilibration for 2 ps, the same force (4.5 nN applied every 10 fs) is applied to K_4_^+^ until it moves out of the filter. The result without the short equilibration is shown in S3 Fig Part E. The different force values are used in the four cases simply to reflect the increased obstacle for the ion crossing the collapsed filter bearing the increasing number of the percolated waters.

Case A shows a fast recovery filter. The two percolated waters, both occupying the upper sites, move less and affect little to the filter recovery process. The collapsed filter readily switches to the conductive conformation as K_4_^+^ moves to the S2 site. Note that if the K^+^ concentration is high, the filter may remain in the conductive conformation as more K^+^ ions permeate through it. Here, with no K^+^ following, the filter spontaneously switches back to the collapsed conformation when K_4_^+^ moves to the S1 site. Case A shows that water molecules located in the upper sites affect little to the filter recovery process.

In Case B, the effects of the percolated waters are noticeable. The two water molecules behind chain D prevent this chain from switching to the conductive conformation as K_4_^+^ moves to the S2 site. Clearly, chain D remains collapsed albeit the bottom percolated water moves up. However, K_4_^+^ still goes through such a nearly conductive filter (three chains conductive, one chain collapsed), and the speed is not reduced much (inferred by the model M1 result in Fig 5). This explains why an asymmetric water pattern is not sufficient to lock the filter inactivated.

When more water molecules percolate behind the filter such as in Case C, the filter recovery depends on the chains having fewer water molecules behind. As the ion moves up, chains A and C both switch to the conductive conformations, leaving the other two chains collapsed. This partially conductive conformation allows ion permeation but with a compromised speed (see the model M3 result in Fig 5), on average, slower than the nearly conductive conformation shown in Case B. However, due to the asymmetric water pattern, there is still the chance that some chains become conductive that reduces the collapsed filter stability. And this is why such a filter is less stable than that having the symmetric water patterns (such as model M6).

When all the water sites are occupied such as in Case D (model M9), theoretically all filter chains are prevented from switching to the conductive conformations. However, there are still the moments when some water molecules are slightly moved. Under the large force, when K_4_^+^ moves to the S2 site, only chain B switches to a roughly conductive conformation, albeit temporarily. The K_4_^+^ ion pushes its way through this nearly collapsed conformation, and subsequently the percolated waters move back to their original locations. Another simulation shows a distorted filter chain as K_4_^+^ moves to the S2 site, reflecting the large obstacles exerted by the percolated waters that prevent the collapsed filter switching to the conductive conformation (S3 Fig Part E). The recoveries of the filters having a large number of the percolated waters, uniformly or nearly uniformly distributed, all look like this (models M6-M9).

Fig 6 demonstrates that ions can go through a nearly conductive (Case B), a partially conductive (Case C), or a nearly collapsed filter (Case D). However, these cases occur when the force acting on the filter ions is large. In reality, the force is small and the collapsed filters with a large number of the percolated waters may remain collapsed over a long period of time. It is not clear how the filter responds over the long period under the condition of small forces. It is also not clear whether the percolated waters move out before the filter recovery takes place, or they can move out during the filter recovery process when multiple ions sequentially move through the filter that gradually switches the filter into the conductive conformation.

## Discussion

Several experiments show the evidence of the buried waters behind the selectivity filter of the KcsA channel. The solid-state NMR spectra show a buried water contacting G79 and L81 in the conductive filter conformation [18], and multiple water sites (buried or nearby) contacting T75, V76, G77, Y78, and G79 in the collapsed filter conformation [6]. In crystallographic studies, Zhou et al. show one water molecule behind a conductive filter chain (Fig 1B) and three water molecules behind a collapsed filter chain (Fig 1C) [7]. Thus the filter must undergo a process during which more water molecules percolate behind it. The simulations show that this process is triggered by the conformational change of filter switched from the conductive to the collapsed conformation, after which water molecules percolate gradually behind the collapsed filter chains. Here, we do not study all the water patterns, instead, we study only several representative patterns because the general conclusions can be drawn from these patterns. Normally, under the similar conditions (such as the ion and the water locations inside the filters are same), the collapsed filter can be stabilized when more water molecules percolate behind it, and the stability is enhanced if the water molecules distribute uniformly or nearly uniformly behind the four filter chains. These simulation results may be utilized to explain some experimental findings.

Experimental evidence shows that hydration of KcsA K^+^ channel can stabilize the collapsed filter conformation. In the solid-state NMR studies, Bhate et al. have found that at low K^+^ concentration, the NMR spectra of the collapsed filter are observed in the hydrated condition but missing in the dehydrated condition [9]. Normally at low K^+^ concentration the filter prefers the collapsed conformation. However, such an expected conformation is missing in the dehydrated environment. Our simulation results may provide some explanations to this finding that the collapsed filters with no or few percolated waters are less stable (Fig 5), that they can easily switch to the conductive conformations (such as the Case A in Fig 6).

The varied modes of the channel open probabilities may due to the dynamic water patterns. The wild-type KcsA channel shows the different modes of the open probabilities varying from the low, to the flickering, and to the high open modes [31, 32]. Here we provide some simulation examples (models M1-M9) that may explain these experimental findings. The different modes of the channel open probabilities may due to the different intervals of the filter switching between the two conformations. And this may result from the different collapsed filter stabilities that are possibly influenced by the dynamic water patterns (Fig 5).

In this study, only short-length simulations are used to study the properties of the transient collapsed filter conformations over the long inactivation period. They demonstrate that the initially formed collapsed filter may not be stable, but it can be stabilized quickly as more water molecules percolate behind it. However, these short-length simulations do not capture the dynamics of the filter near the microsecond time scale as observed in the solid-state NMR studies [33]. Based on the idea that the collapsed filter can have varied stabilities and it can be stabilized gradually, we hypothesize that the slow inactivation observed in the KcsA channel may due to the different stabilities of the collapsed filters over a long period of time. This may result from the environmental disturbances that may affect the percolated water patterns. If the water percolating process extends over a long period of time, the gradual stabilization of the collapsed filter may correlate with the slow inactivation of the KcsA channel. In reality, the collapsed filter can be stabilized due to many factors, and here we have studied only one possible influence, that is from the percolated waters behind the filter. We hypothesize that as the collapsed filter is stabilized gradually, it can inhibit the ion flow that finally leads to the inactivation of the KcsA channel. Currently, the relation between the collapsed filter conformation and the KcsA channel inactivation state is still under debate [34]. Further investigations are needed for the clarification.

## Supporting information

S1 Fig Enlarged plot of Fig 2. Part A, Enlarged plot of Cases 1 and 2. Part B, Enlarged plot of Case 3. Part C, Enlarged plot of Case 4.
(PDF)

S2 Fig Enlarged plot of Fig 3. Part A, Enlarged plot of models M1-M3. Part B, Enlarged plot of models M4-M6. Part C, Enlarged plot of models M7-M10.
(PDF)

S3 Fig Enlarged plot of Fig 6. Part A, Enlarged plot of Case A. Part B, Enlarged plot of Case B. Part C, Enlarged plot of Case C. Part D, Enlarged plot of Case D. Part E, Another filter recovery process of model M9.
(PDF)
