# Supplementary material for: Dynamic water patterns change the stability of the collapsed filter conformation of the KcsA K+ channel
Source: PLoS One. 2017 Oct 19;12(10):e0186789. doi: 10.1371/journal.pone.0186789 (PMC5648213; doi:10.1371/journal.pone.0186789)
Supplement: S1 Fig — Part A, Enlarged plot of Cases 1 and 2. Part B, Enlarged plot of Case 3. Part C, Enlarged plot of Case 4. (PDF) [file pone.0186789.s001.pdf]

Supporting Information: S1 Fig.

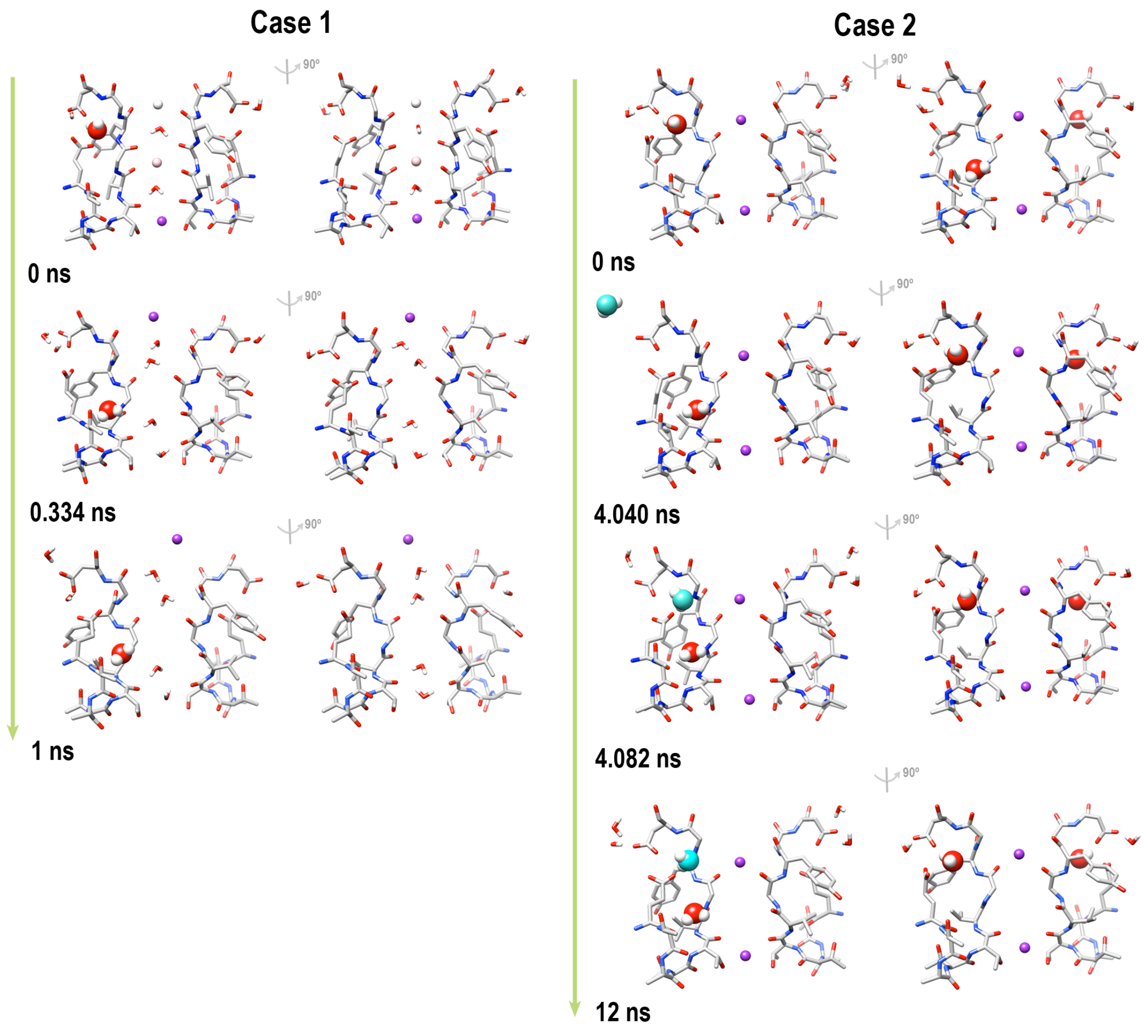

S1 Fig Part A. Enlarged plot of Cases 1 and 2 in Fig 2. The percolated waters are drawn in the sphere representation. The oxygen atoms are colored red for the waters locating behind the filter at the beginning of the simulations. In Case 2, the oxygen atom is colored cyan for the water percolating behind the filter during the simulation.

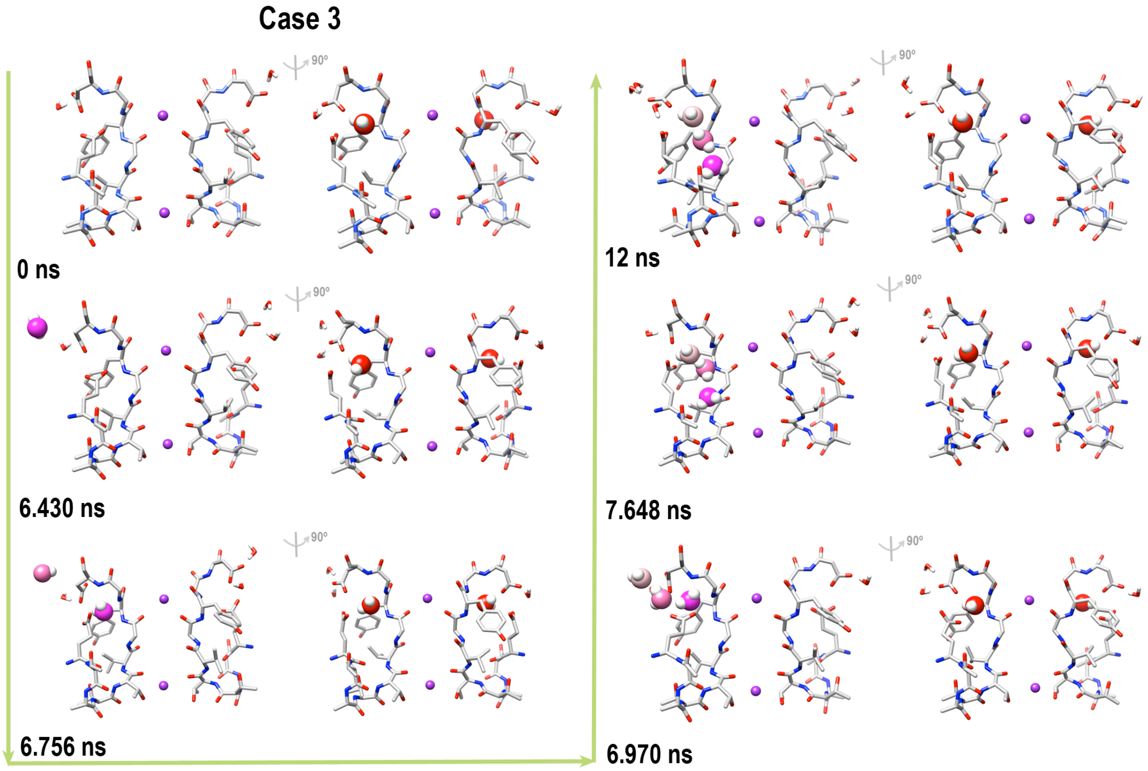

S1 Fig Part B. Enlarged plot of Case 3 in Fig 2. The percolated waters are drawn in the sphere representation. The oxygen atoms are colored red if the waters locate behind the filter at the beginning of the simulations, and are drawn in the different colors if the waters percolate behind the filter during the simulations.

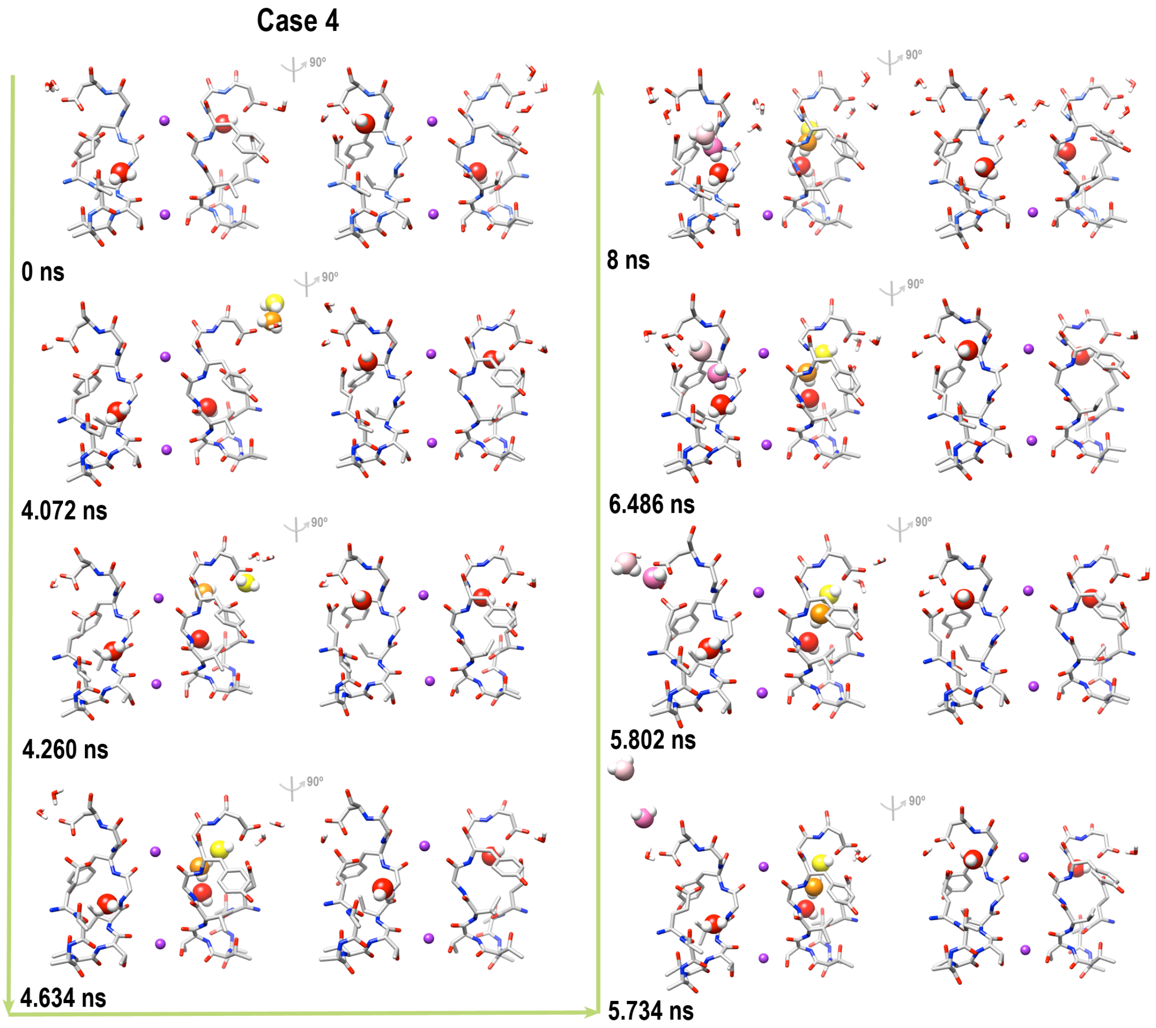

S1 Fig Part C. Enlarged plot of Case 4 in Fig 2. The percolated waters are drawn in the sphere representation. The oxygen atoms are colored red if the waters locate behind the filter at the beginning of the simulations, and are drawn in the different colors if the waters percolate behind the filter during the simulations.
