# Supplementary material for: Dynamic water patterns change the stability of the collapsed filter conformation of the KcsA K+ channel
Source: PLoS One. 2017 Oct 19;12(10):e0186789. doi: 10.1371/journal.pone.0186789 (PMC5648213; doi:10.1371/journal.pone.0186789)
Supplement: S2 Fig — Part A, Enlarged plot of models M1-M3. Part B, Enlarged plot of models M4-M6. Part C, Enlarged plot of models M7-M10. (PDF) [file pone.0186789.s002.pdf]

Supporting Information: S2 Fig.

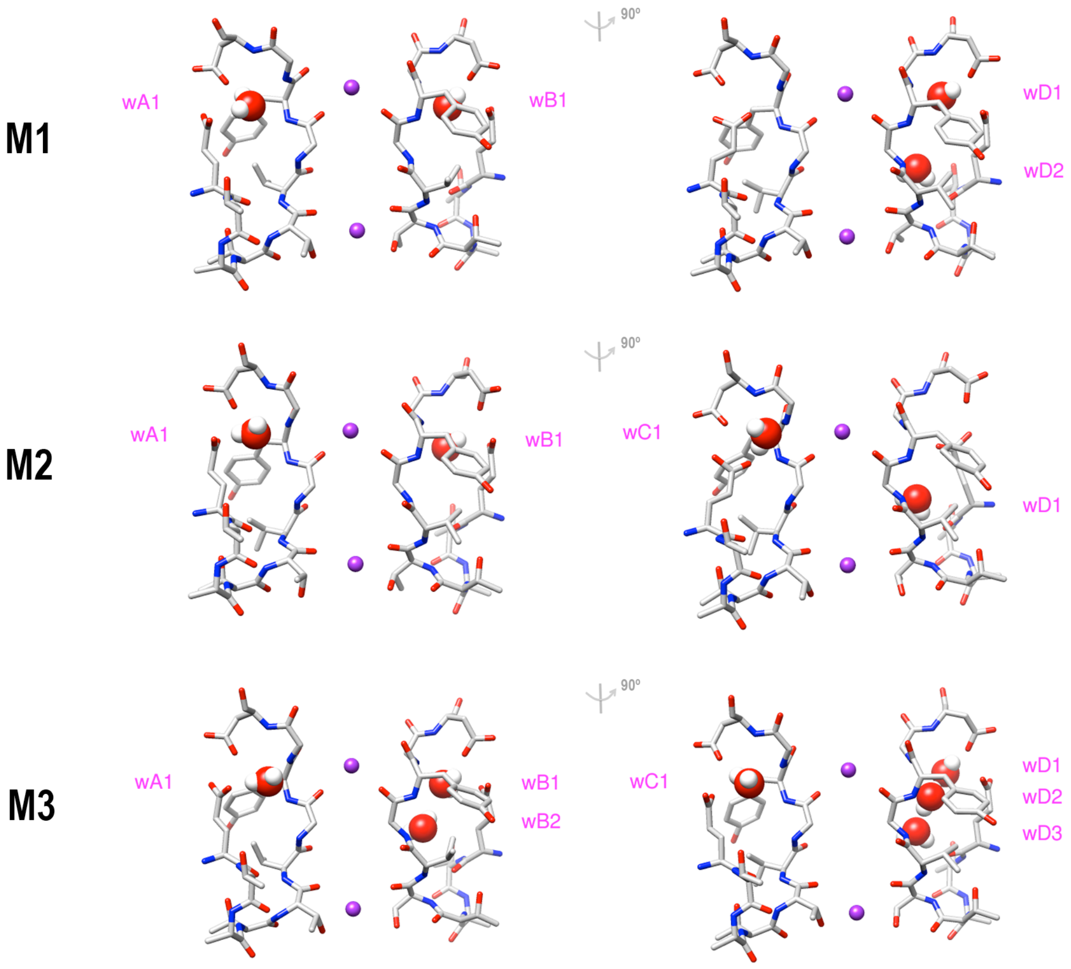

S2 Fig Part A. Enlarged plot of models M1-M3 in Fig 3. The percolated waters are drawn in the sphere representation.

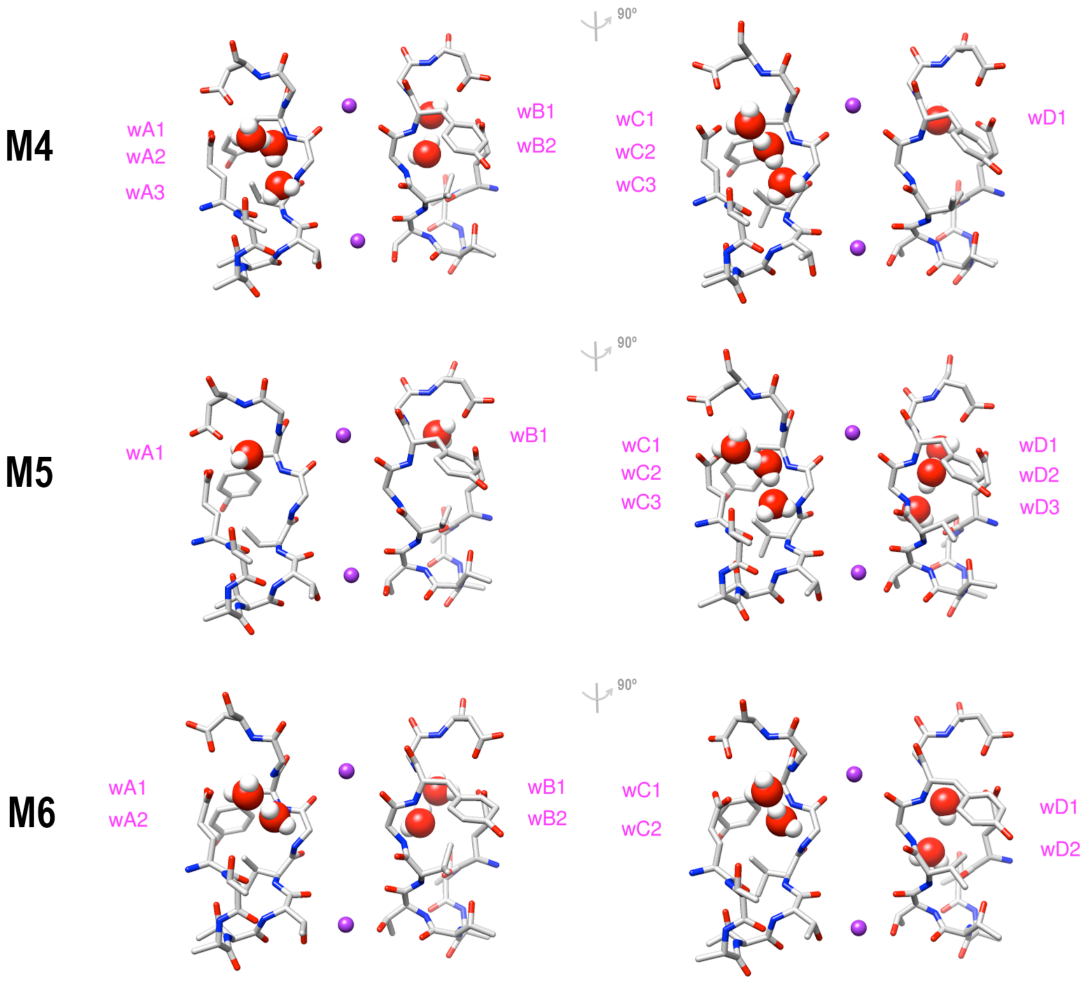

S2 Fig Part B. Enlarged plot of models M4-M6 in Fig 3. The percolated waters are drawn in the sphere representation.

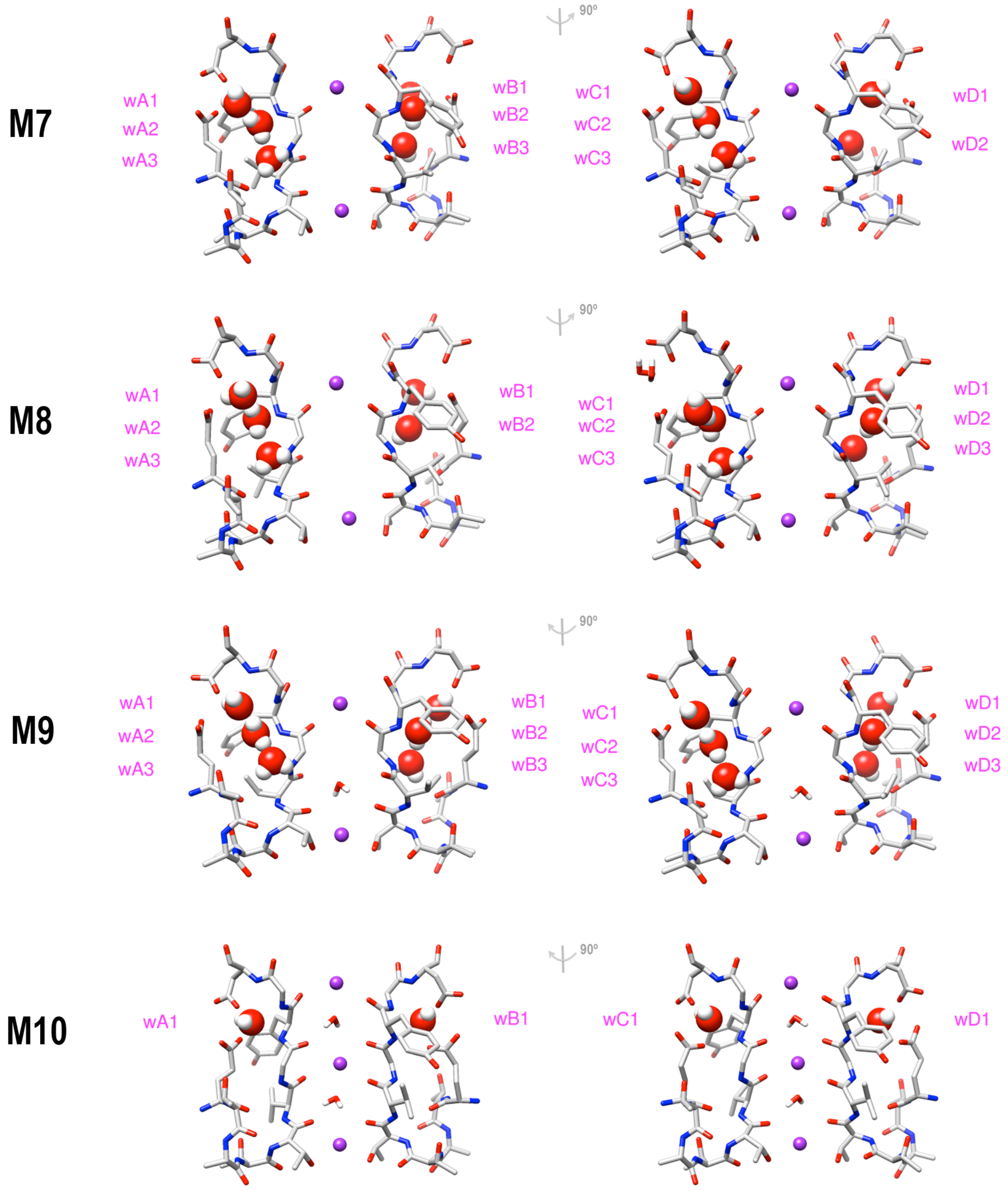

S2 Fig Part C. Enlarged plot of models M7-M10 in Fig 3. The percolated waters are drawn in the sphere representation.
