# Supplementary material for: Dynamic water patterns change the stability of the collapsed filter conformation of the KcsA K+ channel
Source: PLoS One. 2017 Oct 19;12(10):e0186789. doi: 10.1371/journal.pone.0186789 (PMC5648213; doi:10.1371/journal.pone.0186789)
Supplement: S3 Fig — Part A, Enlarged plot of Case A. Part B, Enlarged plot of Case B. Part C, Enlarged plot of Case C. Part D, Enlarged plot of Case D. Part E, Another filter recovery process of model M9. (PDF) [file pone.0186789.s003.pdf]

Supporting Information: S3 Fig.

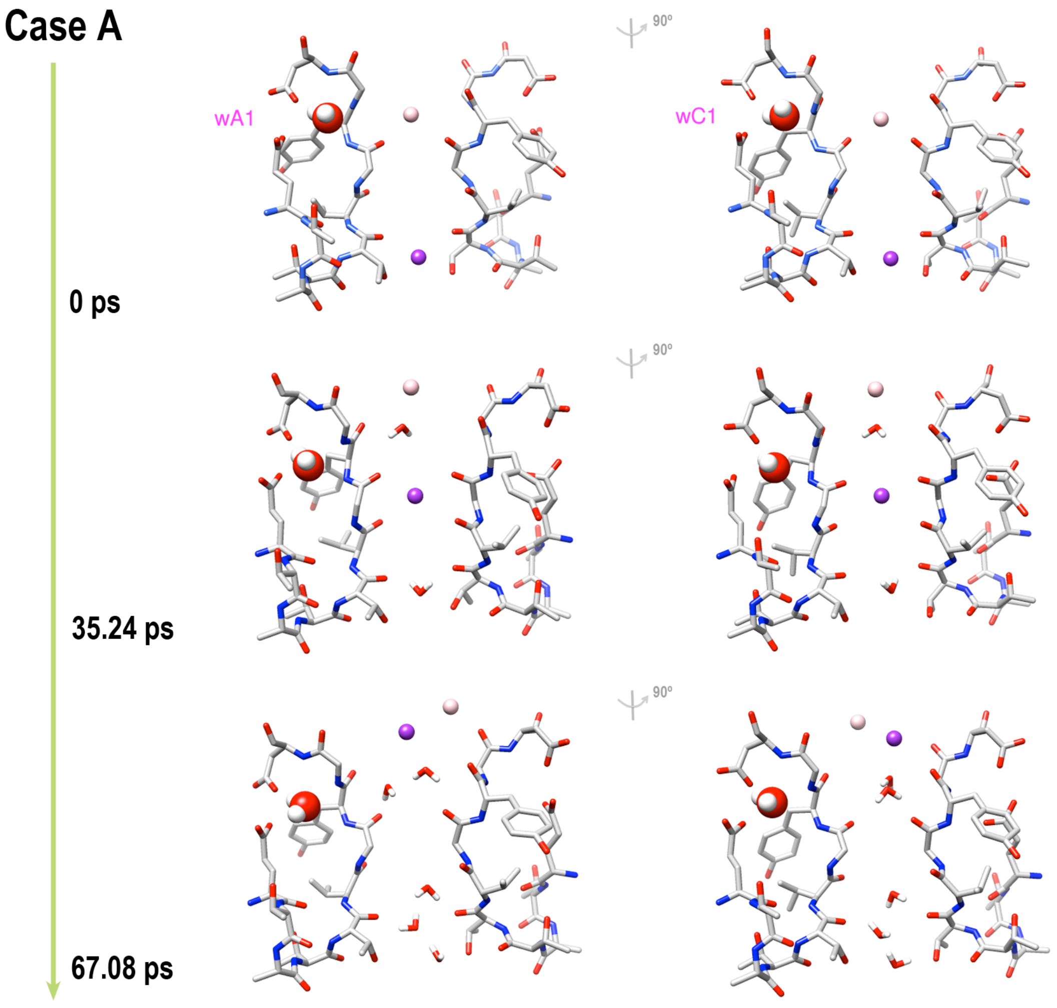

S3 Fig Part A. Enlarged plot of Case A in Fig 6. The percolated waters are drawn in the sphere representation.

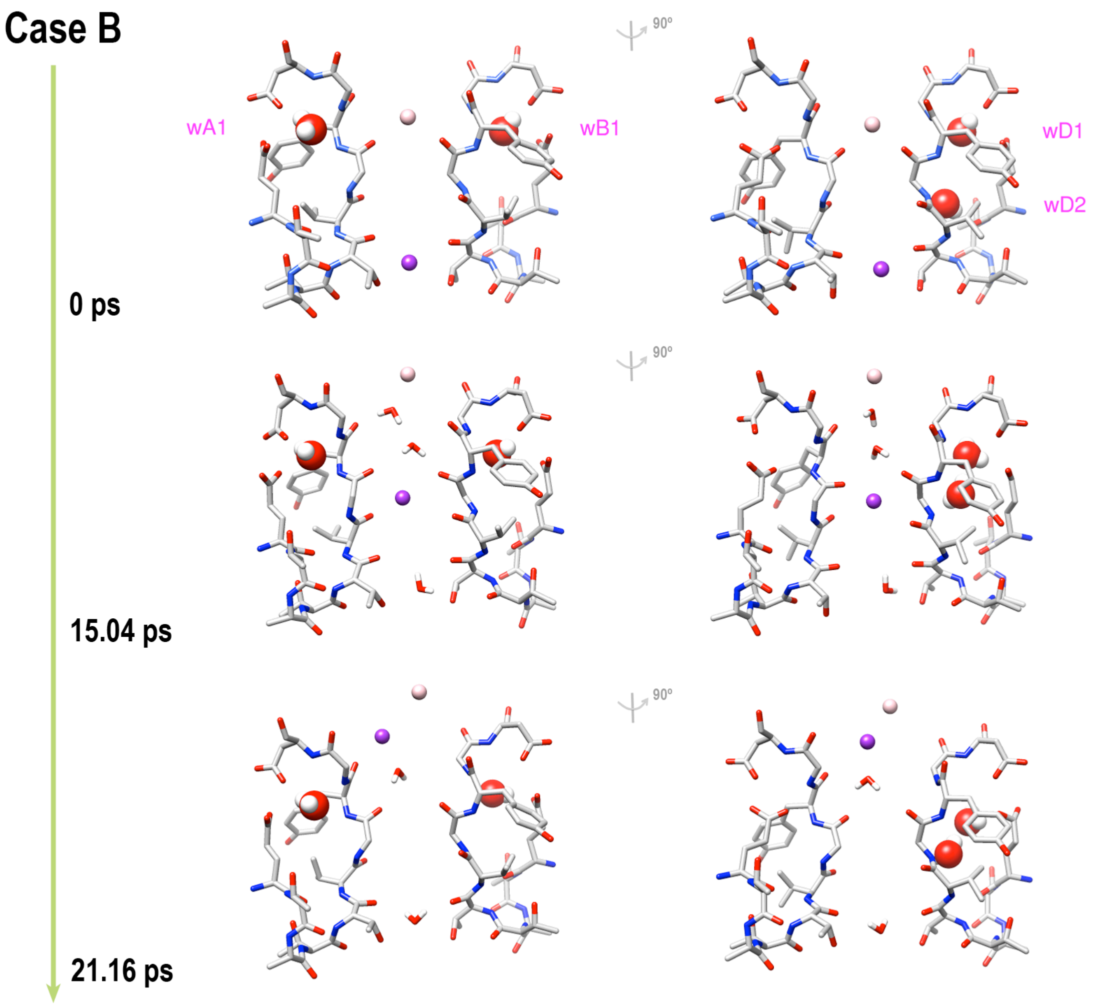

S3 Fig Part B. Enlarged plot of Case B in Fig 6. The percolated waters are drawn in the sphere representation.

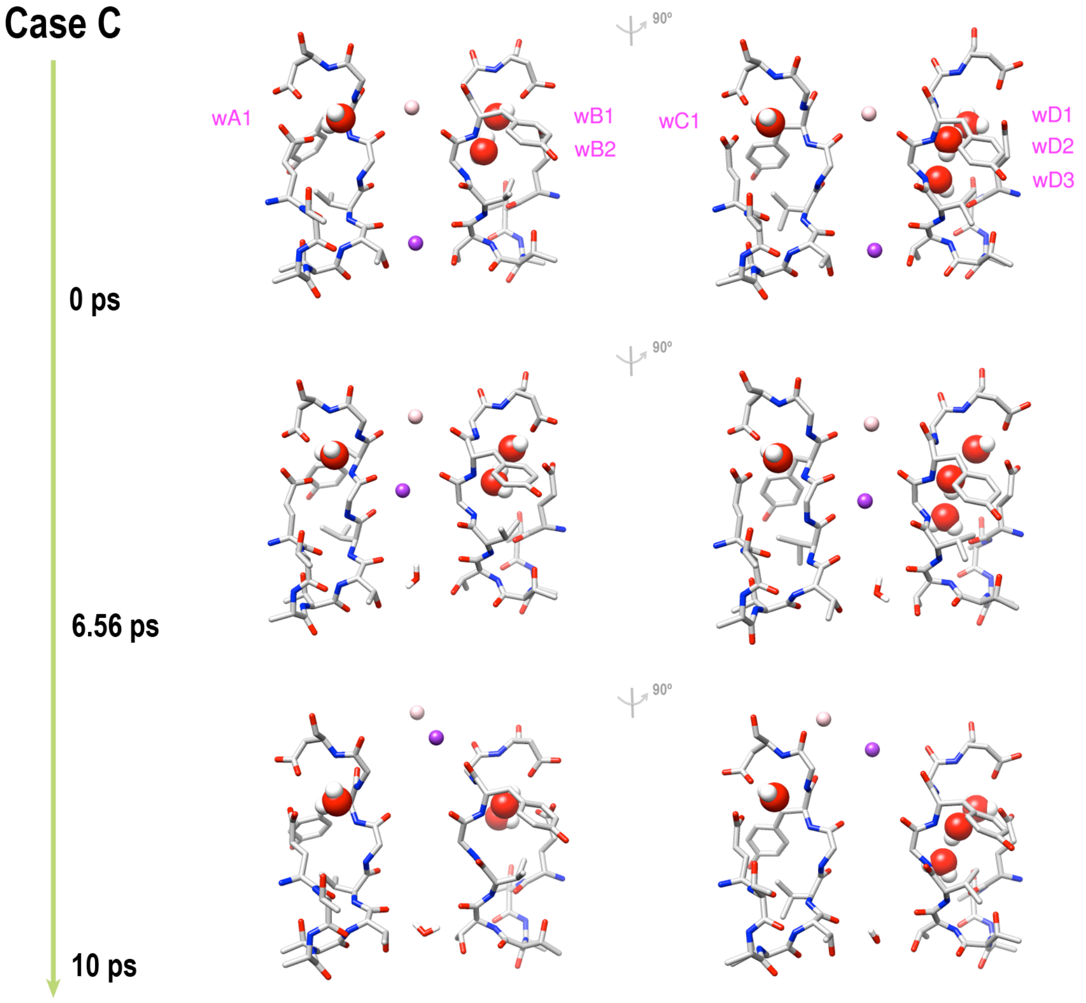

S3 Fig Part C. Enlarged plot of Case C in Fig 6. The percolated waters are drawn in the sphere representation.

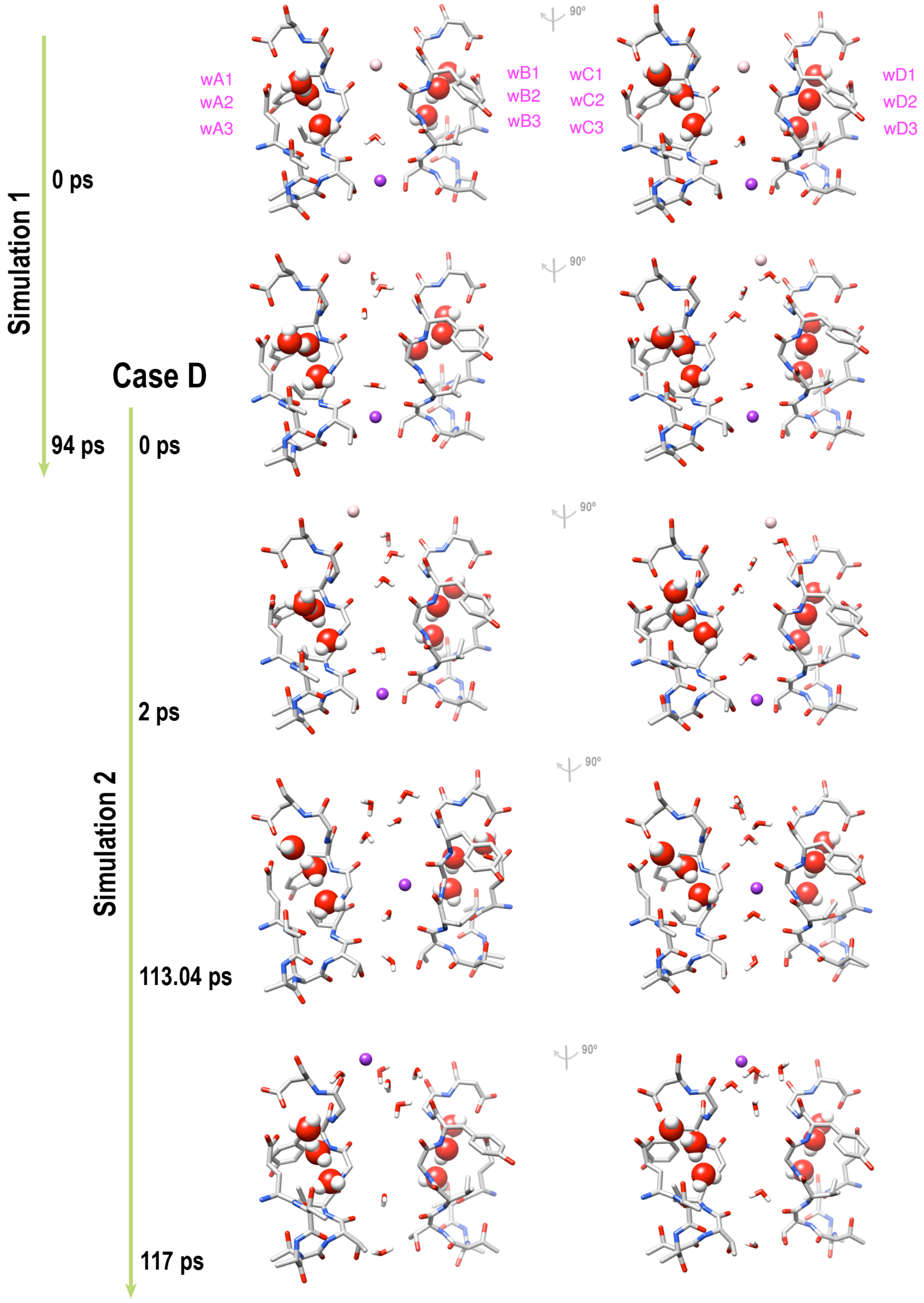

S3 Fig Part D. Enlarged plot of Case D in Fig 6. The percolated waters are drawn in the sphere representation. Case D uses the result from the previous simulation (structure at 94 ps) as the initial configuration. The system is equilibrated shortly for

2 ps before the force is turned on. As shown in this figure, after the 2 ps equilibration, the percolated waters (wB1, wB2, and wB3) relax to their normal locations behind the filter.

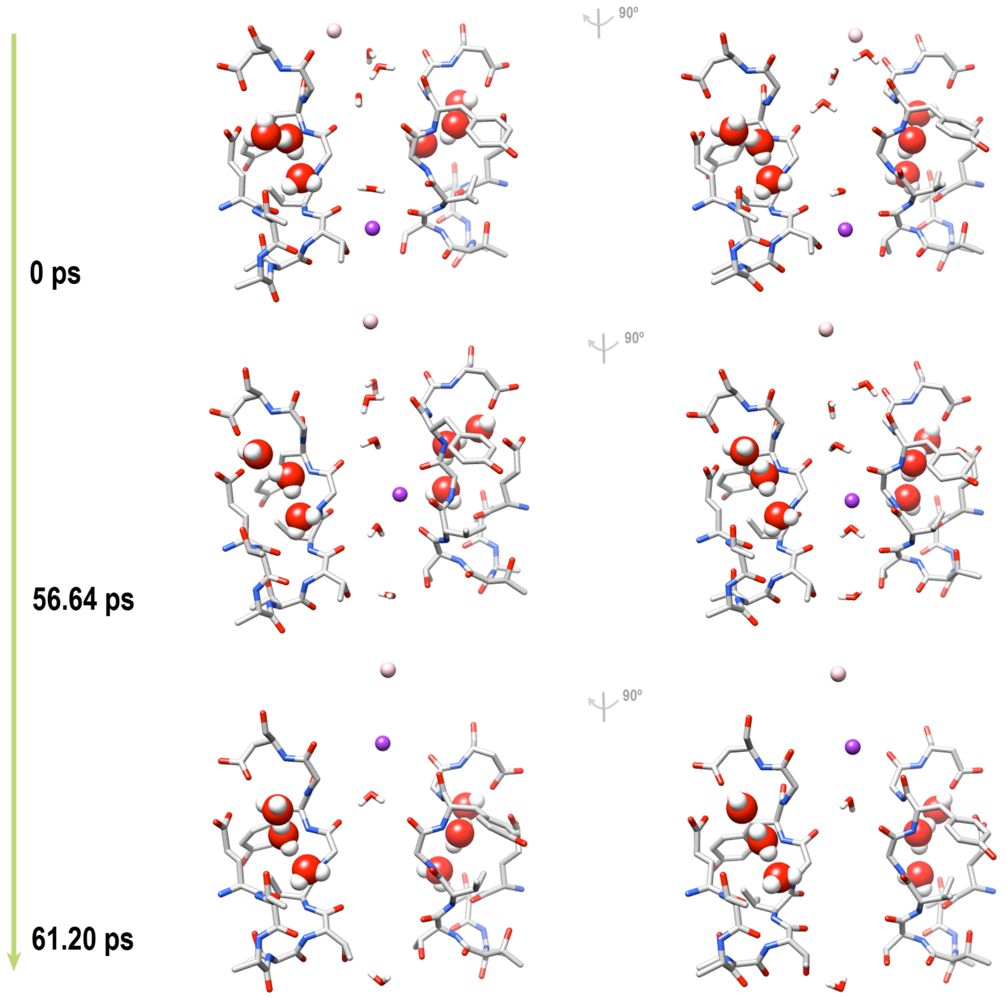

S3 Fig Part E. Another filter recovery process of model M9. Like Case D shown in S3 Fig Part D, the initial configuration uses the result from the previous simulation (structure at 94 ps shown in S3 Fig Part D). This time, the system is not equilibrated, and the force is turned on instantly at 0 ps. All other descriptions are same as for Case D.
